# The ‘double whammy’ of low prevalence in clinical risk prediction

**DOI:** 10.1136/bmjebm-2021-111683

**Published:** 2021-08-13

**Authors:** Thomas R. Fanshawe, Seena Fazel

**Affiliations:** Nuffield Department of Primary Care Health Sciences, University of Oxford, Radcliffe Primary Care Building, Radcliffe Observatory Quarter, Woodstock Road, Oxford OX2 6GG; Department of Psychiatry, University of Oxford, Warneford Hospital, Oxford, OX3 7JX

## Background

Worldwide, around 800,000 people die each year from suicide,[1] which is the leading cause of death in the UK in young adults.[2] Prediction modelling studies have attempted to incorporate demographic, clinical and other factors to identify high-risk individuals so that appropriate interventions can be offered.[3, 4]

This approach has a large literature but has not always been judged successful. In one review, all 35 suicide risk prediction studies assessed were classified as having high risk of bias or insufficient diagnostic accuracy, based on targets of 80% sensitivity and 50% specificity.[5] Others have written of a performance ‘glass ceiling’ in suicide prediction, and even that “risk categorization of individual patients has no role to play in preventing the suicide of psychiatric inpatients”.[6]

In spite of the mortality data quoted above, in most populations risk of death from suicide is low, usually below 1%. This has led to claims about performance of prediction models that appear counterintuitive, such as “suicide prediction models produce accurate overall classification models, but their accuracy of predicting a future event is near 0”, while noting that even in high-risk populations, the positive predictive value (PPV) of many prediction rules may be less than 1%.[3]

We describe two principal reasons why the nature of developing clinical prediction rules in low prevalence scenarios will almost invariably result in concerns about performance, on standards by which this is usually judged. Although we focus primarily on suicide prediction, these points apply more generally to other low prevalence clinical areas, some examples of which are also discussed.

### 1 The effect of low prevalence on sample size

**Figure 1:**
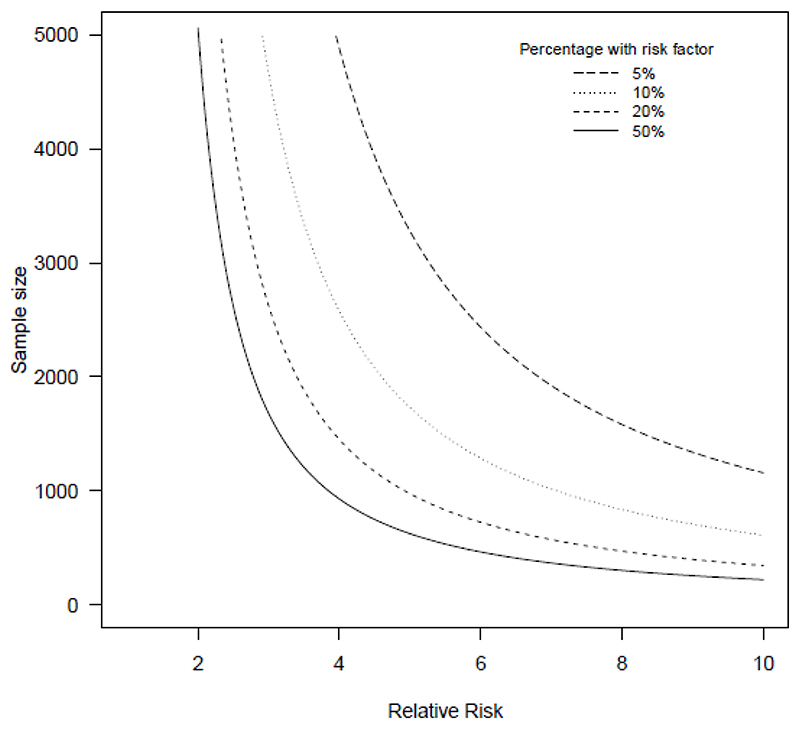
Total sample size required to detect a relative risk of the given size, with 90% power at the 5% significance level, if the prevalence of the outcome in the comparison group is 1% and a certain percentage of the population have the risk factor (5%, 10%, 20% or 50%, as shown by the separate lines).

Low prevalence can have a prohibitive impact on sample size requirements because of the need to observe enough outcome events for model development, as also noted in diagnostic evaluation studies.[7] For example, consider a single risk factor that is present in half of individuals, in a population with outcome prevalence in those without this factor is 1%. To detect a relative risk (RR) of 2 with 90% power (5% significance level), requires a sample size of over 5,000 (equation 8 of [8]). More plausibly, if this risk factor occurs in a minority of individuals (say 10%), this sample size jumps to over 14,000. Figure 1 shows the pattern for larger values of the RR.

Developing prediction models also requires consideration of the effects of using multiple predictors during model selection, overfitting, and validation, which may increase the required sample size manyfold.[9] Although specific methods for determining adequate sample size when developing prediciton models depend on the strength and nature of the associations between the included variables,[9] a good rule of thumb is to include at least ten outcome events for each risk factor examined, to prevent overfitting.[10] Multiplicity of risk factors can become a major issue when the number of predictors is at an extreme, as exemplified by a machine learning study in the US general population that examined 2,978 risk factors in a dataset containing 222 suicide events.[11] Another study based in a single university hospital used 272 risk factors in a dataset with 33 suicide events.[12] The large sample sizes required may make single-site studies infeasible in low prevalence scenarios.

### 2 The effect of low prevalence on predictive performance

The second issue relates to the effect size required of the predictors that would result in levels of prognostic performance deemed acceptable in practice. The population prevalence (p, e.g. 1%) can be considered as the outcome probability if no risk factor information is available. As shown in the Supplementary Material, the relationship between the RR representing the effect of a risk factor (or the combined effect of a set of risk factors), the PPV, the sensitivity (S) and p is then given by

$$RR=\frac{PPV-pS}{p(1-S)}.$$

**Figure 2:**
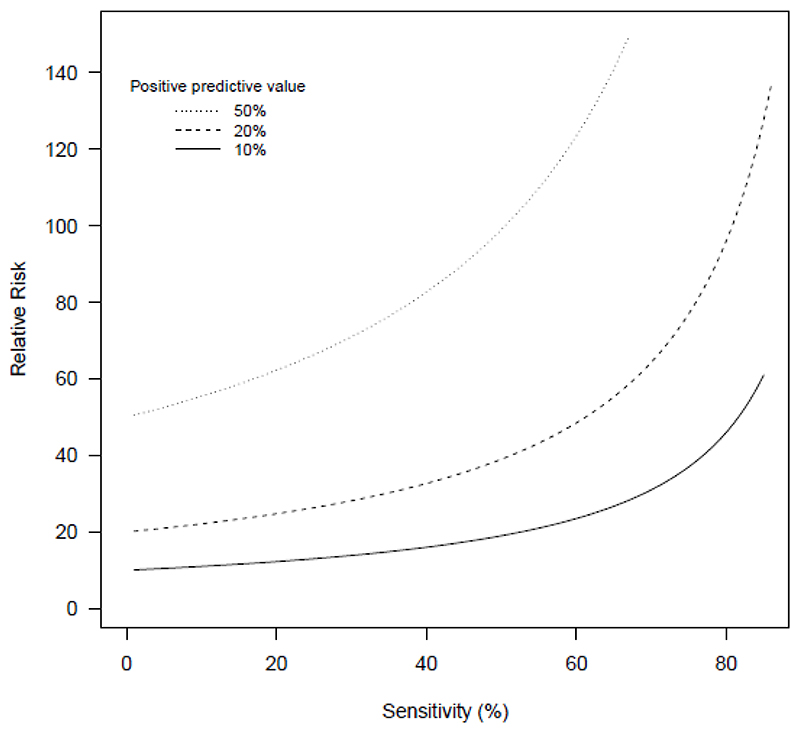
Relative risk as a function of sensitivity, in a population with outcome prevalence of 1%, for different values of the positive predictive value (10%, 20% or 50%, as shown by the separate lines)

This allows the relationship between RR, S and PPV to be illustrated for given prevalence. This can be a useful concept because relative risk as an effect size measure is familiar across many research areas. For the 1% prevalence scenario, Figure 2 plots RR against S for varying PPV. For a PPV of 50%, the RR can never be less than 50, and in the range of sensitivities usually seen as acceptable, it will be higher still. Even for a PPV of 10%, which is higher than that reported for most suicide risk prediction models, the required RR would need to be well over 10.[13]

An alternative measure that is often used to summarise the relationship between prevalence and outcome probability calculated from a risk prediction model is the likelihood ratio. The positive likelihood ratio is defined as Sensitivity/(1-Specificity) and can be interpreted as the change in the odds of the outcome after using the prediction tool. To convert a prevalence 1% to a probability of 10%, 25% or 50% would require the prediction tool to correspond to a positive likelihood ratio of 11, 33 and 99 respectively. Even though it has been noted the likelihood ratio measures themselves may vary with outcome prevalence,[14] the conclusion is the same as that obtained from the RR, as likelihood ratios of this magnitude may appear unachievable.[15]

### Implications

The two issues described in this paper constitute a ‘double whammy’ of low prevalence in risk prediction studies. Effect sizes that are plausible for risk factors require investigations with large sample sizes to reach acceptable statistical power, but predictive performance often appears inadequate even if this sample size is met.

The first issue described may be ameliorated using linked databases of clinical populations, although even these may be insufficient for tools that target less populous subgroups. There is a potential conflict between the size of these databases and the nature of the variables collected: larger routine databases are more likely to contain broad demographic and clinical information than data concerning regular individual-level monitoring, such as symptom change, and are therefore unable to capture short-term changes in symptoms and behaviour that may be associated with increased risk.[16]

The second issue cannot be resolved by increasing sample size alone. Risk prediction using machine learning, often using national datasets, has become popular as an alternative,[17] but the effect of introducing this methodological complexity, as recommended by some authors,[18] appears likely to bring at best an incremental improvement in performance. One review suggested, albeit with uncertainty, that the best performing machine learning methods in suicide prediction might bring performance equivalent to an odds ratio of 12, much less than what would be required under the second condition outlined above.[19] Across a range of epidemiological research areas, relative risks and likelihood ratios of the required magnitude are rarely observed and cannot be realistically expected.[20] While these relative measures may help in developing conceptual understanding of the general issues relating to low prevalence, we recommend that they be presented alongside absolute measures of risk when they are reported in particular studies.[21]

An alternative strategy is to use a more prevalent outcome. In suicide prediction, such an outcome is self harm, which represents a large burden of morbidity, or suicidal ideation. But the uncertainty here is whether risk stratification will lead to changes in clinical practice, if the outcome is not seen as clinically meaningful, and given current estimates of predictive accuracy for self harm outcomes.[22] Although suicide ideation is associated with death from suicide, this association is imperfect and the majority of patients with suicide ideation do not die from suicide.[23]

The considerations outlined in this paper suggest that there should more reasonable expectations about the achievable performance of prediction tools.In suicide risk assessment, there are at least two reasons to consider why a step-up in an individual’s risk from 1% (population prevalence) to 5-10% (modelled: a modest absolute change but large relative change) may be informative when interpreted alongside additional clinical assessment.

Firstly, prediction rules may contain modifiable risk factors that could be targets for treatment, such as suicidal ideation, recent non-adherence to treatment, and comorbidities (for a review of risk factors, see [18]), which allows modifiable risk factors to be embedded into structured risk assessment to improve management and facilitate communication about risk in different healthcare settings. Secondly, the knowledge that an individual is at higher than average risk could be used to underscore safety planning to mitigate that risk.[24] This relatively low-cost intervention, a key component of which is to identify risk factors that may contribute to an individual having elevated risk, has shown promise in reducing suicidal behaviours.[25] However, it must also be acknowledged that a range of other suicide prevention interventions have been demonstrated to be effective, and some of these do not require a specific risk assessment to be made.[26] Some of these are service-level approaches.

## Conclusion

The prevalence of the outcome is a key consideration when planning and carrying out studies of clinical prediction rules. As this article has demonstrated, if the outcome prevalence is low, required sample sizes may become prohibitively large and the calculated risk of individuals identified as those at the highest risk may nevertheless remain quite low on the 0-100% scale in absolute terms.

In the field of suicide prediction, given the likely ceiling on the predictive performance of prediction rules, one implication of this is that prediction rules should not be used in isolation for clinical decision-making and allocation of interventions. Instead, their more appropriate role should be considered as an adjunct for decision-making, and the process of using tools in this way should be scrutinised in respect of their validation, translation into practice, and impact on patient outcomes to ensure that decisions are not harmful for the individual.[27] This requires assessment of outcomes, costs and consequences following the adoption of the prediction tool.[28, 29]

## Supplementary Material

Supplementary Materials
