# Supplementary material for: The ‘double whammy’ of low prevalence in clinical risk prediction
Source: BMJ Evid Based Med. Author manuscript; Available in PMC 2025 Sep 2. (PMC7618060; doi:10.1136/bmjebm-2021-111683)
Supplement: Supplementary Materials [file EMS208263-supplement-Supplementary_Materials.pdf]

Supplementary Material

Derivation of equation in Section 2

For a given risk factor and outcome event, we use the following notation:

	**Outcome positive**	**Outcome negative**	**Total**
**Risk factor positive**	a	b	a+b
**Risk factor negative**	c	d	c+d
**Total**	a+c	b+d	n=a+b+c+d

Additionally write p for the prevalence of the outcome, RR for the relative risk of the risk factor in relation to the outcome event, S for its sensitivity, and PPV and NPV for the positive and negative predictive values, respectively.

By the definitions of these quantities, we have

$$p = \frac{a+c}{n}$$

$$S = \frac{a}{a+c} = \frac{a}{np}$$

$$PPV = \frac{a}{a+b}$$

$$NPV = \frac{d}{c+d}$$

$$RR = \frac{a/(a+b)}{c/(c+d)} = \frac{PPV}{1-NPV}$$

It follows that

$$\begin{aligned} 1 - NPV &= \frac{c}{c+d} \\ &= \frac{n\left(\frac{(a+c)}{n}\right) - a}{n - a\left(\frac{(a+b)}{a}\right)} \\ &= \frac{np-a}{n - a/PPV} \end{aligned}$$

Thus

$$RR = PPV \times \frac{n - a/PPV}{np - a}$$

$$= \frac{nPPV - a}{np - a}$$

$$= \frac{PPV - pS}{p(1-S)}$$
